# Supplementary material for: Different Metabolism and Toxicity of TRANS Fatty Acids, Elaidate and Vaccenate Compared to Cis-Oleate in HepG2 Cells
Source: Int J Mol Sci. 2022 Jun 30;23(13):7298. doi: 10.3390/ijms23137298 (PMC9266973; doi:10.3390/ijms23137298)
Supplement: Supplementary file 1 [file ijms-23-07298-s001.zip › ijms-1713887-supplementary.pdf]

International Journal of *Molecular Sciences*

MDPI

Different metabolism and toxicity of trans fatty acids, elaidate and vaccenate compared to cis-oleate in HepG2 cells

Farkas Sarnyai [1], Éva Kereszturi [1], Kitti Szirmai [1], Judit Mátyási [2], Johanna Iman Al-Hag [2], Tamás Csizmadia [3], Péter Lőw [3], Péter Szelényi [1], Viola Tamási [1], Kinga Tibori [1], Veronika Zámbó [1], Blanka Tóth [2,*] and Miklós Csala [1,*]

1 Semmelweis University, Department of Molecular Biology, Budapest, Hungary
2 Budapest University of Technology and Economics, Department of Inorganic and Analytical Chemistry, Budapest, Hungary
3 Eötvös Loránd University, Department of Anatomy, Cell and Developmental Biology, Budapest, Hungary;
* Correspondence: Email: csala.miklos@med.semmelweis-univ.hu (M.Cs.) and Email: toth.blanka@vbk.bme.hu (B.T.)

Supplementary material

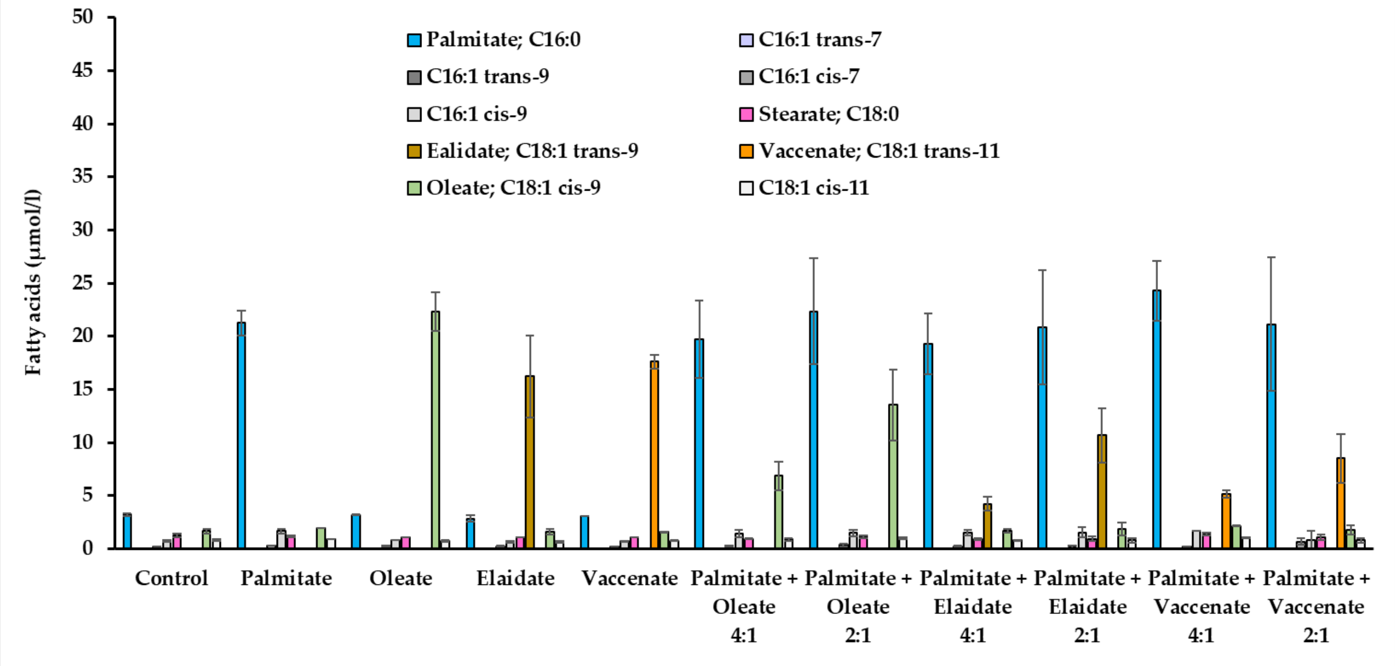

Figure S1. Fatty acid content of the incubation medium of HepG2 cells after 8 h FA supplementations at sub-toxic doses. Cells were treated with BSA-conjugated palmitate, oleate, elaidate or vaccenate (50 μM) or with palmitate (50 μM) and oleate, elaidate or vaccenate at 4:1 or 2:1 molar ratio (12.5 or 25 μM) at 70-80% confluence for 8 h. The concentration of ten relevant fatty acids was measured in the medium by GC-FID after saponification and methylation. Data are shown as mean values ± S.D.; n≥4.

Table S1. Cell-associated fatty acids after 8 h FA supplementations at sub-toxic doses. Cells were treated with BSA-conjugated palmitate, oleate, elaidate or vaccenate (50 µM) or with palmitate (50 µM) and oleate, elaidate or vaccenate at 4:1 or 2:1 molar ratio (12.5 or 25 µM) at 70-80% confluence for 8 h. The amount of ten relevant fatty acids was measured by GC-FID after saponification and methylation. Data were normalized to the total protein content of the samples, and are shown as mean values ± S.D.; n≥4.

Treatment (8 h)	FA content (µg/mg protein)									
	Palmitate C16:0	C16:1 trans-7	C16:1 trans-9	C16:1 cis-7	C16:1 cis-9	Stearate C18:0	Ealidate C18:1 trans-9	Vaccenate C18:1 trans-11	Oleate C18:1 cis-9	C18:1 cis-11
Control	36.0 ± 5.4	n.d.	n.d.	2.7 ± 0.4	17.4 ± 2.7	10.4 ± 1.0	n.d.	n.d.	31.4 ± 5.5	19.8 ± 2.3
Palmitate	51.1 ± 13.9	n.d.	n.d.	2.6 ± 0.6	19.7 ± 5.7	8.7 ± 2.1	n.d.	n.d.	24.7 ± 6.8	18.0 ± 5.0
cis-Oleate	37.9 ± 5.0	n.d.	n.d.	2.9 ± 0.3	15.4 ± 2.0	10.7 ± 1.5	n.d.	n.d.	82.0 ± 11.6	19.8 ± 2.7
trans-Elaidate	32.0 ± 1.0	1.5 ± 0.3	n.d.	2.2 ± 0.3	15.0 ± 1.8	7.9 ± 0.9	44.0 ± 1.1	n.d.	28.4 ± 3.7	17.6 ± 2.4
trans-Vaccenate	38.0 ± 6.1	n.d.	1.4 ± 0.2	2.3 ± 0.3	16.6 ± 3.1	9.7 ± 1.8	n.d.	28.2 ± 6.3	31.1 ± 6.7	19.5 ± 4.1
Palmitate + Oleate (4:1)	64.9 ± 5.7	n.d.	n.d.	3.1 ± 0.3	22.8 ± 1.7	10.9 ± 1.5	n.d.	n.d.	40.1 ± 2.3	21.2 ± 1.5
Palmitate + Oleate (2:1)	65.9 ± 12.4	n.d.	n.d.	3.2 ± 0.4	21.4 ± 3.7	10.6 ± 1.9	n.d.	n.d.	51.4 ± 10.6	20.6 ± 4.0
Palmitate + Elaidate (4:1)	61.8 ± 7.0	0.5 ± 0.1	n.d.	2.9 ± 0.3	23.8 ± 2.6	9.2 ± 1.0	11.7 ± 1.4	n.d.	28.4 ± 3.1	20.1 ± 2.2
Palmitate + Elaidate (2:1)	61.0 ± 13.2	0.8 ± 0.1	n.d.	2.7 ± 0.3	22.5 ± 4.4	8.6 ± 1.4	24.0 ± 5.4	n.d.	27.6 ± 4.7	19.0 ± 3.2
Palmitate + Vaccenate (4:1)	59.7 ± 11.2	n.d.	0.5 ± 0.1	2.4 ± 0.5	21.1 ± 3.9	8.9 ± 1.5	n.d.	6.2 ± 1.2	26.2 ± 4.4	18.4 ± 3.1
Palmitate + Vaccenate (2:1)	63.1 ± 12.5	n.d.	0.8 ± 0.2	2.3 ± 0.5	21.2 ± 4.7	9.3 ± 1.6	n.d.	12.1 ± 2.2	27.9 ± 5.5	18.9 ± 3.9

[1] Not detectable.

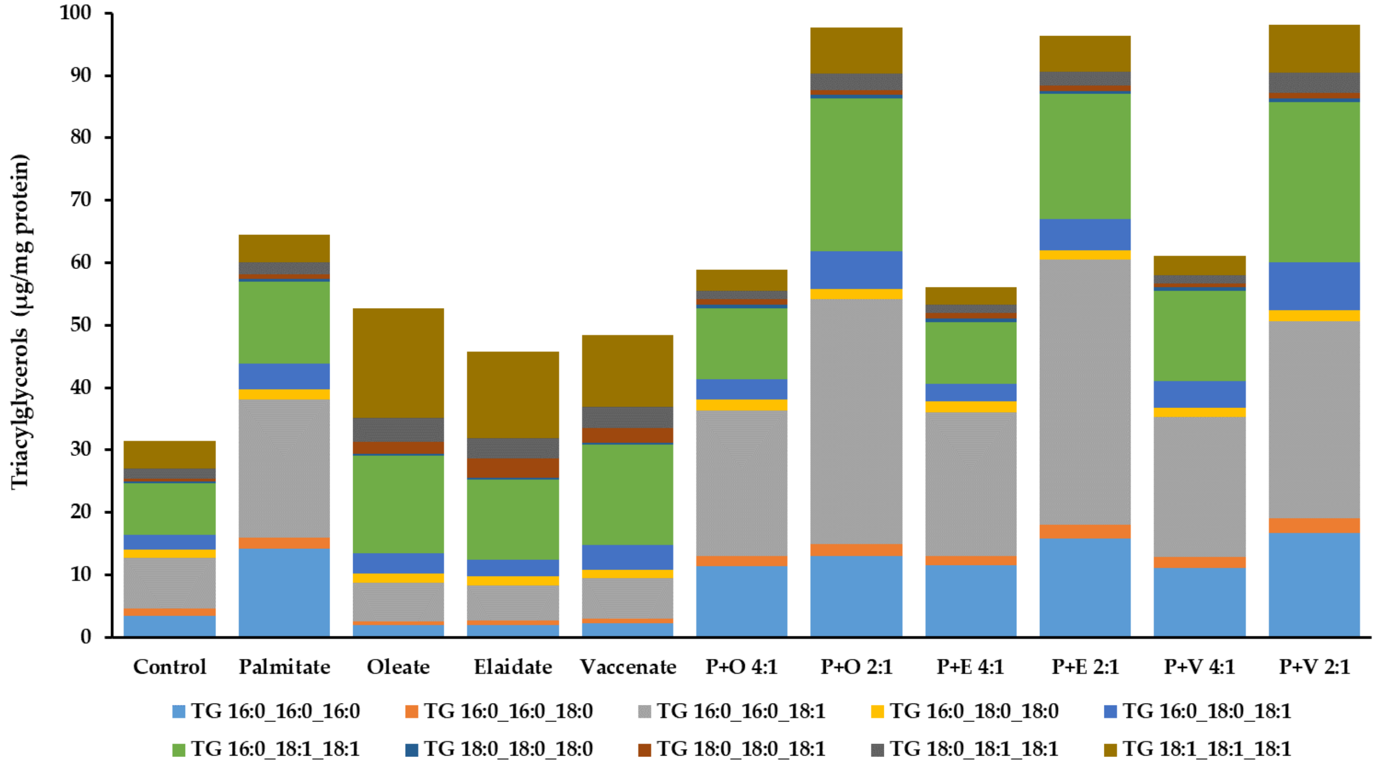

Figure S2. Triacylglycerol content of HepG2 cells after 8 h FA supplementations at sub-toxic doses. Cells were treated with BSA-conjugated palmitate, oleate, elaidate or vaccenate (50 µM) or with palmitate (50 µM) and oleate, elaidate or vaccenate at 4:1 or 2:1 molar ratio (12.5 or 25 µM) at 70-80% confluence for 8 h. The amount of relevant triacylglycerols was measured by LC-MS/MS. Data were normalized to the total protein content of the samples, and are shown as mean values of four independent experiments (see the mean and S.D. values in Table S2).

Table S2. Triacylglycerol content of HepG2 cells after 8 h FA supplementations at sub-toxic doses. Cells were treated with BSA-conjugated palmitate, oleate, elaidate or vaccenate (50 μM) or with palmitate (50 μM) and oleate, elaidate or vaccenate at 4:1 or 2:1 molar ratio (12.5 or 25 μM) at 70-80% confluence for 8 h. The amount of relevant triacylglycerols was measured by LC-MS/MS. Data were normalized to the total protein content of the samples, and are shown as mean values ± S.D.; n≥4.

Treatment (8 h)	Triacylglycerol content (μg/mg protein)									
	TG 16:0_16:0_16:0	TG 16:0_16:0_18:0	TG 16:0_16:0_18:1	TG 16:0_18:0_18:0	TG 16:0_18:0_18:1	TG 16:0_18:1_18:1	TG 18:0_18:0_18:0	TG 18:0_18:0_18:1	TG 18:0_18:1_18:1	TG 18:1_18:1_18:1
Control	3.4 ± 0.5	1.2 ± 0.2	8.0 ± 2.3	1.4 ± 0.3	2.4 ± 0.7	8.2 ± 2.3	0.3 ± 0.1	0.4 ± 0.1	1.7 ± 0.5	4.3 ± 1.5
Palmitate	14.1 ± 4.2	1.9 ± 0.5	22.0 ± 8.1	1.7 ± 0.2	4.1 ± 1.1	13.2 ± 3.3	0.4 ± 0.1	0.7 ± 0.2	1.9 ± 0.5	4.4 ± 1.2
cis-Oleate	1.9 ± 0.3	0.7 ± 0.1	6.2 ± 1.5	1.5 ± 0.2	3.2 ± 1.1	15.7 ± 5.2	0.3 ± 0.1	1.9 ± 0.3	3.9 ± 1.4	17.5 ± 6.3
trans-Elaidate	1.9 ± 0.2	0.7 ± 0.1	5.7 ± 1.3	1.4 ± 0.3	2.7 ± 0.5	12.8 ± 2.3	0.2 ± 0.1	3.1 ± 0.6	3.3 ± 0.6	13.9 ± 2.5
trans-Vaccenate	2.2 ± 0.2	0.8 ± 0.1	6.4 ± 1.0	1.4 ± 0.3	4.0 ± 0.9	16.0 ± 3.4	0.3 ± 0.1	2.3 ± 0.4	3.5 ± 0.5	11.4 ± 2.2
Palmitate + Oleate (4:1)	11.4 ± 2.1	1.5 ± 0.2	23.4 ± 5.7	1.8 ± 0.3	3.2 ± 0.6	11.4 ± 2.2	0.6 ± 0.2	0.8 ± 0.1	1.4 ± 0.3	3.4 ± 0.7
Palmitate + Oleate (2:1)	13.0 ± 3.6	1.8 ± 0.4	39.4 ± 13.4	1.5 ± 0.3	6.1 ± 1.5	24.6 ± 5.9	0.5 ± 0.1	0.9 ± 0.1	2.6 ± 0.6	7.4 ± 2.0
Palmitate + Elaidate (4:1)	11.5 ± 2.5	1.6 ± 0.3	23.0 ± 6.0	1.8 ± 0.3	2.8 ± 0.7	9.9 ± 2.6	0.6 ± 0.2	0.9 ± 0.1	1.2 ± 0.3	2.9 ± 0.8
Palmitate + Elaidate (2:1)	15.8 ± 3.8	2.1 ± 0.4	42.5 ± 13.2	1.6 ± 0.3	5.0 ± 1.0	20.0 ± 4.1	0.6 ± 0.2	0.9 ± 0.1	2.2 ± 0.4	5.7 ± 1.2
Palmitate + Vaccenate (4:1)	11.1 ± 2.0	1.7 ± 0.2	22.5 ± 4.5	1.5 ± 0.3	4.2 ± 0.6	14.5 ± 2.0	0.5 ± 0.1	0.7 ± 0.2	1.3 ± 0.2	3.1 ± 0.6
Palmitate + Vaccenate (2:1)	16.7 ± 4.6	2.3 ± 0.5	31.5 ± 10.2	1.8 ± 0.3	7.7 ± 2.4	25.6 ± 8.3	0.7 ± 0.2	0.8 ± 0.2	3.3 ± 1.0	7.7 ± 2.5

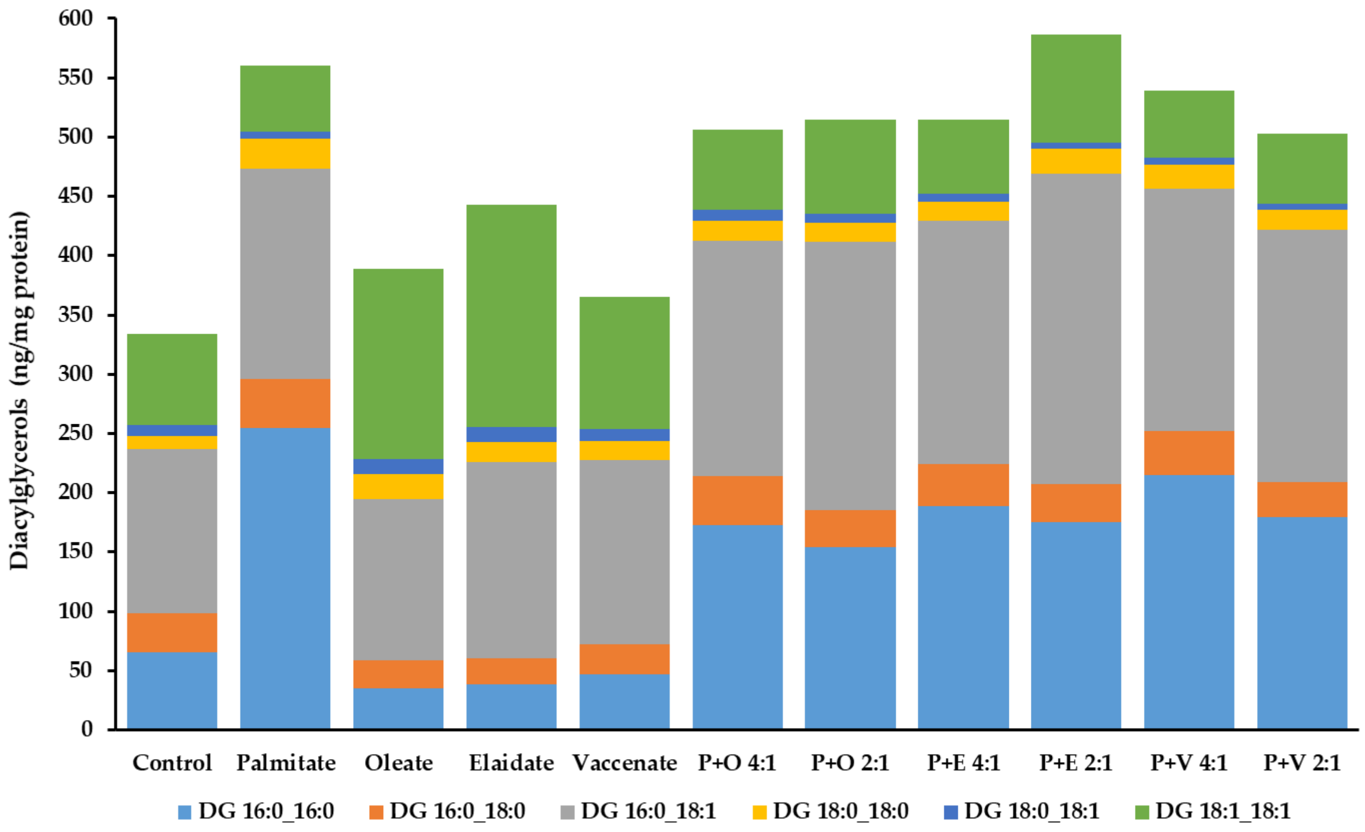

Figure S3. Diacylglycerol content of HepG2 cells after 8 h FA supplementations at sub-toxic doses. Cells were treated with BSA-conjugated palmitate, oleate, elaidate or vaccenate (50 μM) or with palmitate (50 μM) and oleate, elaidate or vaccenate at 4:1 or 2:1 molar ratio (12.5 or 25 μM) at 70-80% confluence for 8 h. The amount of 6 relevant diacylglycerols (columns) was measured by LC-MS/MS. Data were normalized to the total protein content of the samples, and are shown as mean values of at least four independent experiments (see the mean and S.D. values in Table S3).

Table S3. Diacylglycerol content of HepG2 cells after 8 h FA supplementations at sub-toxic doses. Cells were treated with BSA-conjugated palmitate, oleate, elaidate or vaccenate (50 μM) or with palmitate (50 μM) and oleate, elaidate or vaccenate at 4:1 or 2:1 molar ratio (12.5 or 25 μM) at 70-80% confluence for 8 h. The amount of relevant diacylglycerols was measured by LC-MS/MS. Data were normalized to the total protein content of the samples, and are shown as mean values ± S.D.; n≥4.

Treatment (8 h)	Diacylglycerol content (ng/mg protein)					
	DG 16:0_16:0	DG 16:0_18:0	DG 16:0_18:1	DG 18:0_18:0	DG 18:0_18:1	DG 18:1_18:1
Control	65.8 ± 11.3	32.7 ± 17.1	138.7 ± 30.8	10.5 ± 4.8	9.0 ± 4.8	77.1 ± 11.6
Palmitate	254.3 ± 37.9	41.4 ± 6.7	177.4 ± 50.1	25.5 ± 5.8	5.8 ± 1.5	55.9 ± 7.6
cis-Oleate	34.8 ± 11.5	23.6 ± 13.3	136.5 ± 34.3	20.7 ± 4.9	12.8 ± 4.7	160.5 ± 34.2
trans-Elaidate	38.8 ± 11.7	21.2 ± 8.3	166.3 ± 33.0	16.3 ± 5.7	13.2 ± 4.2	187.2 ± 29.0
trans-Vaccenate	47.0 ± 11.1	25.4 ± 12.8	155.2 ± 38.2	16.2 ± 5.7	10.1 ± 3.0	111.1 ± 23.8
Palmitate + Oleate (4:1)	172.9 ± 27.9	41.5 ± 22.1	198.0 ± 17.4	16.8 ± 3.5	9.6 ± 6.0	67.6 ± 11.4
Palmitate + Oleate (2:1)	153.9 ± 30.7	31.1 ± 12.4	226.4 ± 60.5	16.3 ± 6.1	7.9 ± 3.3	78.8 ± 11.9
Palmitate + Elaidate (4:1)	189.1 ± 22.9	35.3 ± 14.4	205.4 ± 27.4	15.5 ± 3.5	6.8 ± 3.9	62.3 ± 5.6
Palmitate + Elaidate (2:1)	175.1 ± 39.5	31.8 ± 8.9	262.0 ± 62.4	21.7 ± 4.7	4.7 ± 2.6	90.9 ± 15.5
Palmitate + Vaccenate (4:1)	215.0 ± 45.7	37.5 ± 12.7	203.6 ± 59.1	20.8 ± 5.3	5.6 ± 2.4	56.3 ± 9.9
Palmitate + Vaccenate (2:1)	179.3 ± 40.6	29.9 ± 7.8	212.3 ± 54.5	17.4 ± 5.6	4.7 ± 2.7	59.3 ± 11.5

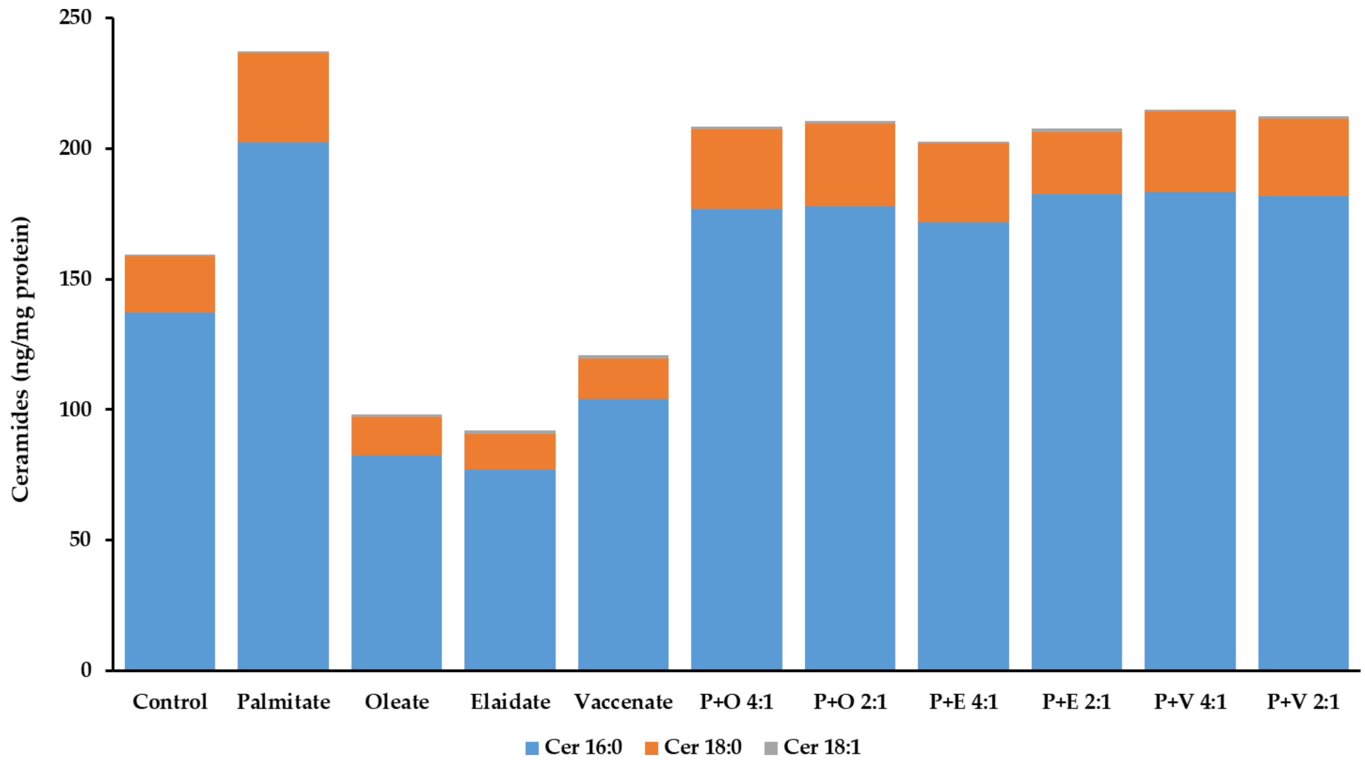

Figure S4. Ceramide content of HepG2 cells after 8 h FA supplementations at sub-toxic doses. Cells were treated with BSA-conjugated palmitate, oleate, elaidate or vaccenate (50 μM) or with palmitate (50 μM) and oleate, elaidate or vaccenate at 4:1 or 2:1 molar ratio (12.5 or 25 μM) at 70-80% confluence for 8 h. The amount of thee relevant ceramides was measured by LC-MS/MS. Data were normalized to the total protein content of the samples, and are shown as mean values of at least four independent experiments (see the mean and S.D. values in Table S4).

Table S4. Ceramide content of HepG2 cells after 8 h FA supplementations at sub-toxic doses. Cells were treated with BSA-conjugated palmitate, oleate, elaidate or vaccenate (50 μM) or with palmitate (50 μM) and oleate, elaidate or vaccenate at 4:1 or 2:1 molar ratio (12.5 or 25 μM) at 70-80% confluence for 8 h. The amount of 3 relevant ceramides was measured by LC-MS/MS. Data were normalized to the total protein content of the samples, and are shown as mean values ± S.D.; n≥4.

**Treatment (8 h)**	**Ceramide content (ng/mg protein)**		
	**Cer 16:0**	**Cer 18:0**	**Cer 18:1**
Control	137.1 ± 20.0	21.6 ± 1.3	0.9 ± 0.1
Palmitate	202.1 ± 11.9	34.2 ± 9.5	0.9 ± 0.2
cis-Oleate	82.4 ± 13.2	14.8 ± 3.8	1.1 ± 0.4
trans-Elaidate	77.1 ± 16.8	13.5 ± 3.3	1.3 ± 0.5
trans-Vaccenate	103.9 ± 26.2	15.5 ± 1.8	1.3 ± 0.4
Palmitate + Oleate (4:1)	176.8 ± 39.3	30.7 ± 3.3	0.9 ± 0.2
Palmitate + Oleate (2:1)	177.7 ± 25.8	32.0 ± 6.7	1.0 ± 0.5
Palmitate + Elaidate (4:1)	171.8 ± 30.3	30.0 ± 6.7	0.6 ± 0.2
Palmitate + Elaidate (2:1)	182.3 ± 34.7	24.1 ± 8.1	1.1 ± 0.4
Palmitate + Vaccenate (4:1)	183.3 ± 30.4	30.7 ± 9.8	0.9 ± 0.3
Palmitate + Vaccenate (2:1)	181.7 ± 31.2	29.6 ± 4.0	1.2 ± 0.5

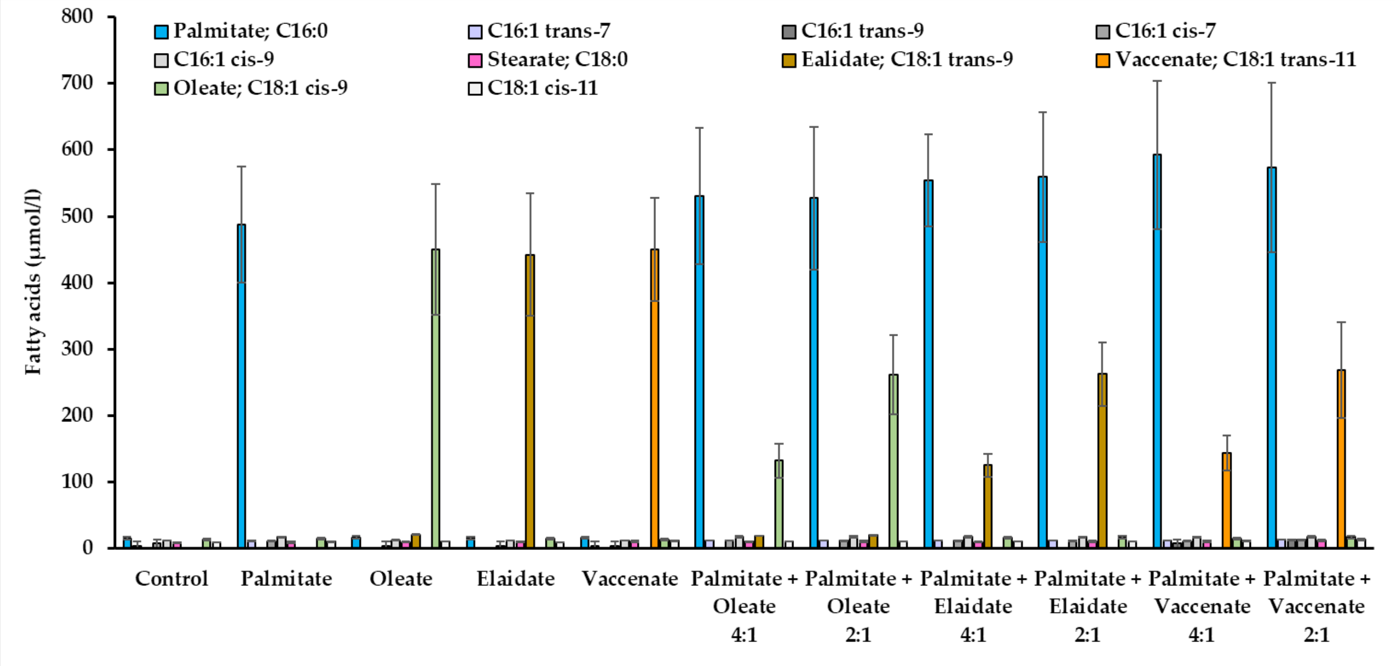

Figure S5. Fatty acid content of the incubation medium of HepG2 cells after 8 h FA supplementations at toxic doses. Cells were treated with BSA-conjugated palmitate, oleate, elaidate or vaccenate (800 μM) or with palmitate (800 μM) and oleate, elaidate or vaccenate at 4:1 or 2:1 molar ratio (200 or 400 μM) at 70-80% confluence for 8 h. The concentration of ten relevant fatty acids was measured in the medium by GC-FID after saponification and methylation. Data are shown as mean values ± S.D.; n≥4.

Table S5. Cell-associated fatty acids after FA supplementations at toxic doses. Cells were treated with BSA-conjugated palmitate, oleate, elaidate or vaccenate (800 μM) or with palmitate (800 μM) and oleate, elaidate or vaccenate at 4:1 or 2:1 molar ratio (200 or 400 μM) at 70-80% confluence for 4 or 8 h. The amount of 10 relevant fatty acids was measured by GC-FID after saponification and methylation. Data were normalized to the total protein content of the samples, and are shown as mean values ± S.D.; n≥4.

Treatment (4 h)	FA content (μg/mg protein)									
	Palmitate C16:0	C16:1 trans-7	C16:1 trans-9	C16:1 cis-7	C16:1 cis-9	Stearate C18:0	Ealidate C18:1 trans-9	Vaccenate C18:1 trans-11	Oleate C18:1 cis-9	C18:1 cis-11
Control	33.6 ± 11.5	n.d. [1]	n.d.	2.5 ± 0.4	13.0 ± 3.6	11.0 ± 2.3	n.d.	n.d.	26.3 ± 3.3	14.7 ± 3.6
Palmitate	229.0 ± 37.1	n.d.	n.d.	3.5 ± 0.3	25.5 ± 4.8	17.3 ± 4.0	n.d.	n.d.	31.4 ± 3.2	18.5 ± 2.2
cis-Oleate	32.9 ± 5.2	n.d.	n.d.	3.3 ± 0.7	11.5 ± 2.0	12.4 ± 3.4	n.d.	n.d.	237.7 ± 30.3	14.6 ± 3.4
trans-Elaidate	26.9 ± 6.2	2.3 ± 0.7	n.d.	2.2 ± 0.4	11.1 ± 3.3	10.2 ± 3.6	208.9 ± 62.6	n.d.	24.8 ± 5.0	12.8 ± 2.9
trans-Vaccenate	33.2 ± 5.2	n.d.	3.8 ± 0.6	2.6 ± 0.3	12.4 ± 2.6	11.7 ± 2.9	n.d.	213.0 ± 39.6	27.5 ± 4.0	15.8 ± 3.3
Palmitate + Oleate (4:1)	215.3 ± 39.5	n.d.	n.d.	3.2 ± 0.7	20.6 ± 5.1	15.9 ± 3.5	n.d.	n.d.	55.7 ± 7.9	15.8 ± 5.7
Palmitate + Oleate (2:1)	184.1 ± 16.4	n.d.	n.d.	2.7 ± 0.4	19.0 ± 3.1	14.4 ± 3.1	n.d.	n.d.	78.2 ± 6.4	13.7 ± 2.6
Palmitate + Elaidate (4:1)	210.0 ± 42.7	0.7 ± 0.1	n.d.	3.1 ± 0.6	21.3 ± 3.7	16.3 ± 6.0	40.7 ± 6.9	n.d.	28.9 ± 4.1	15.7 ± 2.5
Palmitate + Elaidate (2:1)	183.6 ± 25.4	1.0 ± 0.1	n.d.	2.8 ± 0.2	18.4 ± 2.1	13.7 ± 3.3	71.7 ± 10.3	n.d.	25.8 ± 1.3	14.1 ± 2.1
Palmitate + Vaccenate (4:1)	204.3 ± 50.0	n.d.	1.1 ± 0.2	2.8 ± 0.4	20.6 ± 5.2	15.1 ± 5.0	n.d.	37.5 ± 9.3	27.1 ± 3.9	15.3 ± 1.5
Palmitate + Vaccenate (2:1)	198.0 ± 42.3	n.d.	1.7 ± 0.4	2.6 ± 0.3	17.3 ± 2.9	14.4 ± 4.1	n.d.	74.2 ± 18.2	26.1 ± 3.2	14.4 ± 1.7

Treatment (8 h)	FA content (μg/mg protein)									
	Palmitate C16:0	C16:1 trans-7	C16:1 trans-9	C16:1 cis-7	C16:1 cis-9	Stearate C18:0	Ealidate C18:1 trans-9	Vaccenate C18:1 trans-11	Oleate C18:1 cis-9	C18:1 cis-11
Control	36.1 ± 19.4	n.d.	n.d.	2.6 ± 0.7	12.8 ± 4.9	10.5 ± 2.2	n.d.	n.d.	24.9 ± 3.3	14.6 ± 4.0
Palmitate	409.7 ± 80.7	n.d.	n.d.	4.8 ± 1.0	34.3 ± 8.7	24.4 ± 10.1	n.d.	n.d.	34.8 ± 7.3	21.7 ± 1.7
cis-Oleate	30.2 ± 3.1	n.d.	n.d.	3.5 ± 0.5	9.4 ± 1.8	11.0 ± 2.6	n.d.	n.d.	362.6 ± 48.4	13.3 ± 3.3
trans-Elaidate	24.9 ± 2.6	4.1 ± 0.5	n.d.	2.1 ± 0.4	9.8 ± 2.5	8.8 ± 1.9	370.8 ± 108.6	n.d.	23.8 ± 4.0	12.3 ± 3.4
trans-Vaccenate	30.7 ± 2.8	n.d.	6.5 ± 0.6	2.3 ± 0.3	10.3 ± 2.3	10.5 ± 2.4	n.d.	360.8 ± 77.8	24.5 ± 3.2	14.7 ± 3.5
Palmitate + Oleate (4:1)	353.3 ± 86.0	n.d.	n.d.	3.7 ± 0.3	25.8 ± 2.5	18.3 ± 3.0	n.d.	n.d.	74.4 ± 10.9	16.5 ± 3.6
Palmitate + Oleate (2:1)	294.7 ± 75.5	n.d.	n.d.	3.0 ± 0.3	23.2 ± 4.4	16.1 ± 3.3	n.d.	n.d.	109.7 ± 16.9	13.5 ± 2.5
Palmitate + Elaidate (4:1)	352.9 ± 97.1	1.4 ± 0.4	n.d.	3.5 ± 0.3	27.6 ± 3.2	18.6 ± 5.1	71.3 ± 18.9	n.d.	29.7 ± 3.6	17.4 ± 3.0
Palmitate + Elaidate (2:1)	253.5 ± 65.9	1.7 ± 0.3	n.d.	2.4 ± 0.4	20.0 ± 4.1	12.9 ± 3.8	105.1 ± 23.7	n.d.	21.6 ± 3.2	12.2 ± 2.5
Palmitate + Vaccenate (4:1)	311.3 ± 63.2	n.d.	2.0 ± 0.4	3.1 ± 0.2	24.5 ± 3.9	17.0 ± 2.5	n.d.	62.0 ± 19.0	26.9 ± 1.8	16.2 ± 2.6
Palmitate + Vaccenate (2:1)	273.4 ± 47.7	n.d.	2.8 ± 0.5	2.5 ± 0.3	18.4 ± 5.1	14.5 ± 2.3	n.d.	109.3 ± 24.1	22.8 ± 4.2	13.5 ± 4.0

[1] Not detectable.

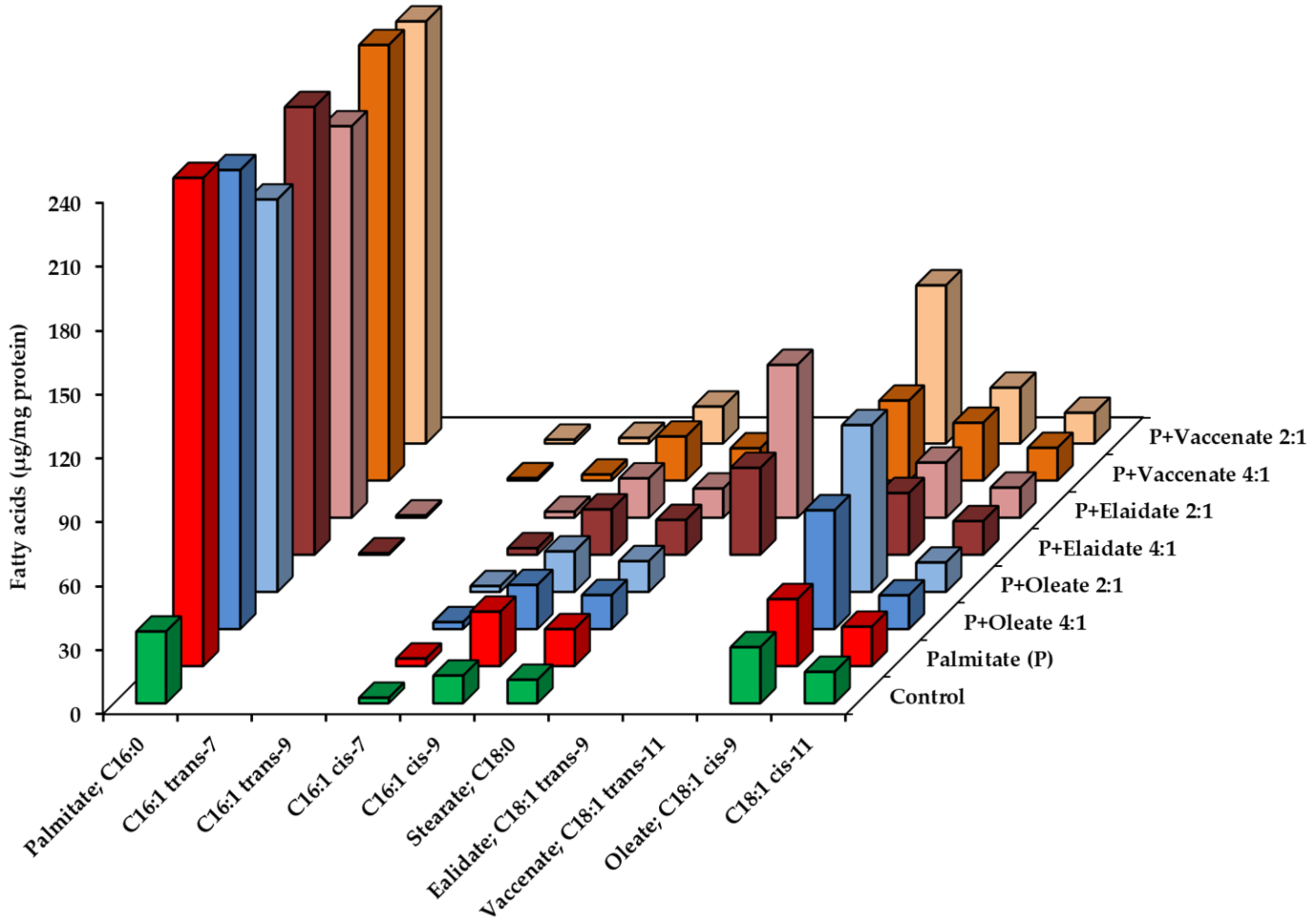

Figure S6. Fatty acid profile of HepG2 cells supplemented with high concentration palmitate and a monounsaturated fatty acid for 4 h. Cells were treated with BSA-conjugated palmitate (800 µM) and oleate, elaidate or vaccenate at 4:1 or 2:1 molar ratio (200 or 400 µM) at 70-80% confluence for 4 h. The amount of relevant fatty acids was measured by GC-FID after saponification and methylation. Data were normalized to the total protein content of the samples, and are shown as mean values of at least five independent experiments.

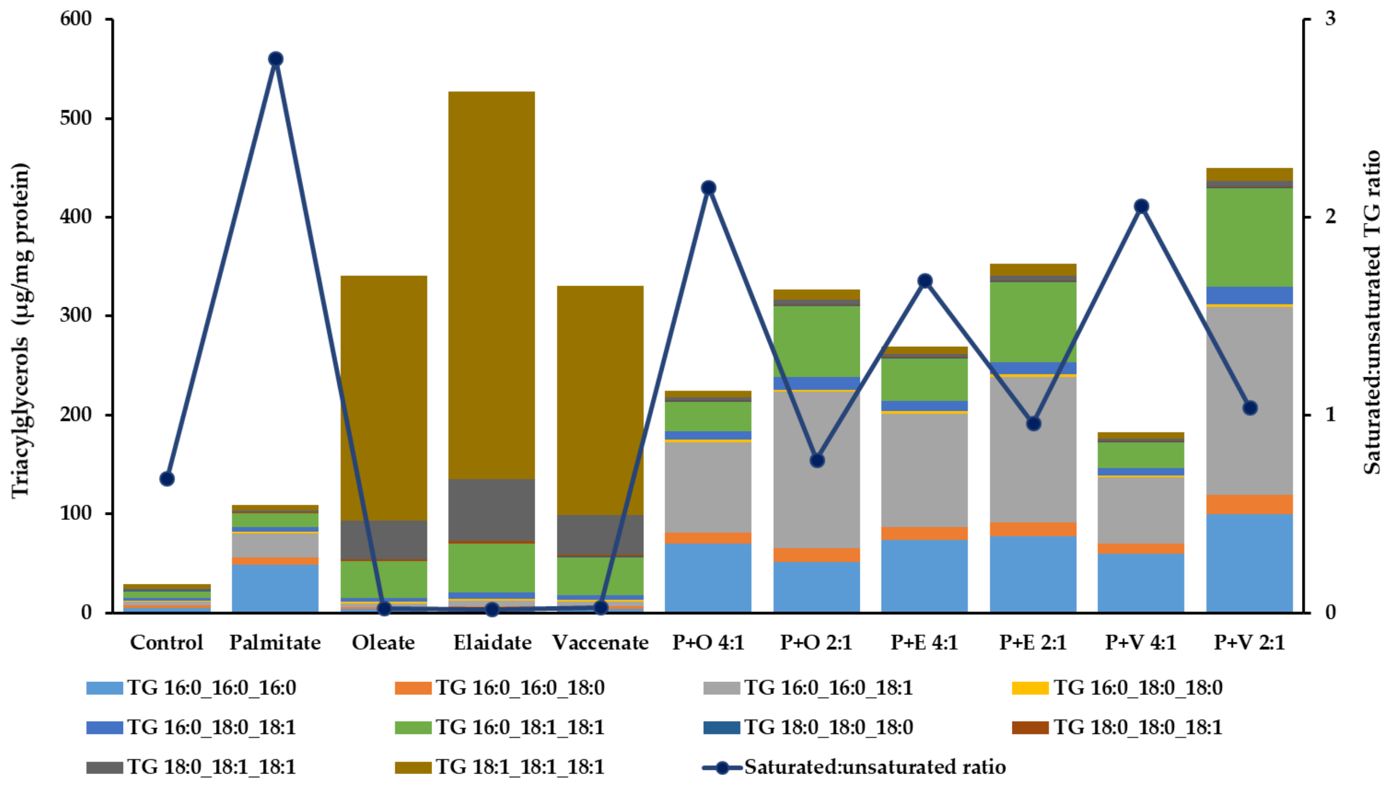

Figure S7. Triacylglycerol content of hepatoma cells in lipotoxicity after 4 h. Cells were treated with BSA-conjugated palmitate, oleate, elaidate or vaccenate (800 μM) or with palmitate (800 μM) and oleate, elaidate or vaccenate at 4:1 or 2:1 molar ratio (200 or 400 μM) at 70-80% confluence for 4 h. The amount of relevant triacylglycerols (columns) was measured by LC-MS/MS. Data were normalized to the total protein content of the samples, and are shown as mean values of four independent experiments (see the mean and S.D. values in Table S2). The ratio of the mean values for fully saturated types (TG 16:0_16:0_16:0, TG 16:0_16:0_18:0, TG 16:0_18:0_18:0 and TG 18:0_18:0_18:0) and those containing at least two unsaturated chains (TG 16:0_18:1_18:1, TG 18:0_18:1_18:1 and TG 18:1_18:1_18:1) was calculated for each treatment (full circles connected with a continuous line for better visibility, right axis).

Table S6. Triacylglycerol content of hepatoma cells in lipotoxicity. Cells were treated with BSA-conjugated palmitate, oleate, elaidate or vaccenate (800 μM) or with palmitate (800 μM) and oleate, elaidate or vaccenate at 4:1 or 2:1 molar ratio (200 or 400 μM) at 70-80% confluence for 4 or 8 h. The amount of relevant triacylglycerols was measured by LC-MS/MS. Data were normalized to the total protein content of the samples, and are shown as mean values ± S.D.; n≥5.

	Triacylglycerol content (μg/mg protein)									
**Treatment (4 h)**	**TG 16:0_16:0_16:0**	**TG 16:0_16:0_18:0**	**TG 16:0_16:0_18:1**	**TG 16:0_18:0_18:0**	**TG 16:0_18:0_18:1**	**TG 16:0_18:1_18:1**	**TG 18:0_18:0_18:0**	**TG 18:0_18:0_18:1**	**TG 18:0_18:1_18:1**	**TG 18:1_18:1_18:1**
Control	4.7 ± 2.1	2.9 ± 1.3	3.5 ± 1.3	1.3 ± 0.5	2.4 ± 1.1	7.1 ± 2.6	0.3 ± 0.2	0.4 ± 0.2	1.8 ± 0.7	4.6 ± 1.6
Palmitate	48.9 ± 16.6	7.2 ± 1.6	24.1 ± 5.0	2.2 ± 0.7	4.8 ± 1.8	13.1 ± 4.3	0.4 ± 0.2	0.7 ± 0.4	2.5 ± 1.1	5.4 ± 2.0
cis-Oleate	3.5 ± 1.3	2.4 ± 0.8	3.9 ± 1.5	1.1 ± 0.3	4.5 ± 2.9	36.6 ± 9.4	0.3 ± 0.1	1.9 ± 0.5	39.2 ± 9.5	247.3 ± 58.6
trans-Elaidate	4.3 ± 2.3	2.7 ± 1.5	5.2 ± 3.7	1.6 ± 1.2	6.6 ± 6.8	49.2 ± 22.7	0.2 ± 0.2	3.1 ± 1.1	62.5 ± 23.9	391.1 ± 133.4
trans-Vaccenate	3.9 ± 2.1	2.7 ± 1.6	4.9 ± 2.4	1.4 ± 0.8	5.2 ± 3.9	38.1 ± 17.5	0.3 ± 0.2	2.3 ± 1.2	39.9 ± 23.2	231.8 ± 133.0
Palmitate + Oleate (4:1)	69.7 ± 27.2	11.8 ± 5.4	91.4 ± 33.5	2.6 ± 1.4	8.4 ± 4.1	29.7 ± 12.2	0.6 ± 0.3	0.8 ± 0.5	2.9 ± 1.5	6.8 ± 3.2
Palmitate + Oleate (2:1)	51.2 ± 14.8	13.7 ± 6.9	158.2 ± 39.8	2.1 ± 1.0	13.5 ± 6.5	71.6 ± 21.9	0.5 ± 0.2	0.9 ± 0.5	4.5 ± 1.9	11.1 ± 3.9
Palmitate + Elaidate (4:1)	73.6 ± 24.4	12.8 ± 6.4	114.9 ± 34.8	2.7 ± 1.4	10.1 ± 4.2	42.9 ± 12.4	0.6 ± 0.3	0.9 ± 0.5	3.4 ± 1.2	7.1 ± 2.4
Palmitate + Elaidate (2:1)	77.7 ± 31.7	13.7 ± 8.1	147.0 ± 57.1	2.4 ± 0.9	12.7 ± 5.6	81.1 ± 19.0	0.6 ± 0.2	0.9 ± 0.4	4.6 ± 1.1	12.7 ± 2.4
Palmitate + Vaccenate (4:1)	59.5 ± 30.1	10.1 ± 5.8	67.1 ± 35.5	2.3 ± 1.2	7.1 ± 3.6	26.5 ± 11.7	0.5 ± 0.3	0.7 ± 0.4	2.6 ± 1.3	6.1 ± 2.9
Palmitate + Vaccenate (2:1)	99.9 ± 54.6	19.6 ± 13.8	189.9 ± 99.5	2.7 ± 1.5	18.0 ± 10.0	99.2 ± 40.3	0.7 ± 0.4	0.8 ± 0.4	5.5 ± 2.1	13.6 ± 5.0

	Triacylglycerol content (μg/mg protein)									
**Treatment (8 h)**	**TG 16:0_16:0_16:0**	**TG 16:0_16:0_18:0**	**TG 16:0_16:0_18:1**	**TG 16:0_18:0_18:0**	**TG 16:0_18:0_18:1**	**TG 16:0_18:1_18:1**	**TG 18:0_18:0_18:0**	**TG 18:0_18:0_18:1**	**TG 18:0_18:1_18:1**	**TG 18:1_18:1_18:1**
Control	7.0 ± 4.4	5.1 ± 3.1	4.8 ± 2.5	2.9 ± 2.0	3.9 ± 2.1	11.2 ± 6.2	0.7 ± 0.4	0.8 ± 0.5	3.0 ± 1.7	7.8 ± 4.3
Palmitate	75.4 ± 27.6	13.0 ± 2.4	50.2 ± 4.6	4.3 ± 1.2	8.6 ± 2.2	21.2 ± 7.4	1.1 ± 0.3	1.2 ± 0.4	3.6 ± 1.4	7.2 ± 2.4
cis-Oleate	3.3 ± 1.0	2.2 ± 0.8	4.6 ± 1.7	1.4 ± 0.5	8.1 ± 4.0	58.3 ± 18.6	0.4 ± 0.1	3.2 ± 1.0	68.4 ± 20.5	403.3 ± 116.7
trans-Elaidate	3.1 ± 1.2	2.2 ± 0.9	3.4 ± 1.5	1.3 ± 0.6	6.3 ± 3.1	44.4 ± 16.3	0.3 ± 0.1	3.1 ± 1.1	64.9 ± 26.5	381.8 ± 154.5
trans-Vaccenate	3.7 ± 1.4	2.8 ± 1.1	4.4 ± 1.6	1.5 ± 0.6	7.1 ± 3.3	49.2 ± 15.6	0.4 ± 0.2	3.0 ± 1.0	55.4 ± 21.6	313.2 ± 113.2
Palmitate + Oleate (4:1)	46.0 ± 31.6	15.5 ± 11.8	139.2 ± 106.6	2.7 ± 2.3	13.2 ± 10.1	46.0 ± 33.8	0.6 ± 0.5	0.8 ± 0.6	3.6 ± 2.7	7.3 ± 5.5
Palmitate + Oleate (2:1)	57.1 ± 24.8	25.6 ± 11.5	258.3 ± 105.0	3.4 ± 1.7	28.7 ± 15.1	139.9 ± 78.0	0.7 ± 0.4	1.3 ± 0.8	7.3 ± 4.5	18.9 ± 12.3
Palmitate + Elaidate (4:1)	62.4 ± 23.0	21.4 ± 11.3	177.6 ± 107.9	4.3 ± 2.0	20.5 ± 11.9	87.2 ± 51.0	1.0 ± 0.5	1.3 ± 0.6	6.0 ± 3.3	13.9 ± 7.6
Palmitate + Elaidate (2:1)	70.5 ± 40.6	33.9 ± 20.0	329.1 ± 196.3	4.9 ± 2.4	44.9 ± 24.2	244.7 ± 132.7	1.2 ± 0.6	1.9 ± 0.9	12.7 ± 6.3	36.5 ± 18.6
Palmitate + Vaccenate (4:1)	44.1 ± 16.4	17.3 ± 11.1	139.3 ± 100.0	3.7 ± 2.4	19.3 ± 13.7	82.6 ± 61.7	0.9 ± 0.6	1.4 ± 1.0	6.3 ± 4.5	13.7 ± 9.9
Palmitate + Vaccenate (2:1)	78.7 ± 26.1	26.1 ± 10.4	271.7 ± 91.1	4.7 ± 1.7	35.7 ± 15.9	206.0 ± 74.3	1.1 ± 0.5	1.7 ± 0.7	11.4 ± 3.9	31.4 ± 12.0

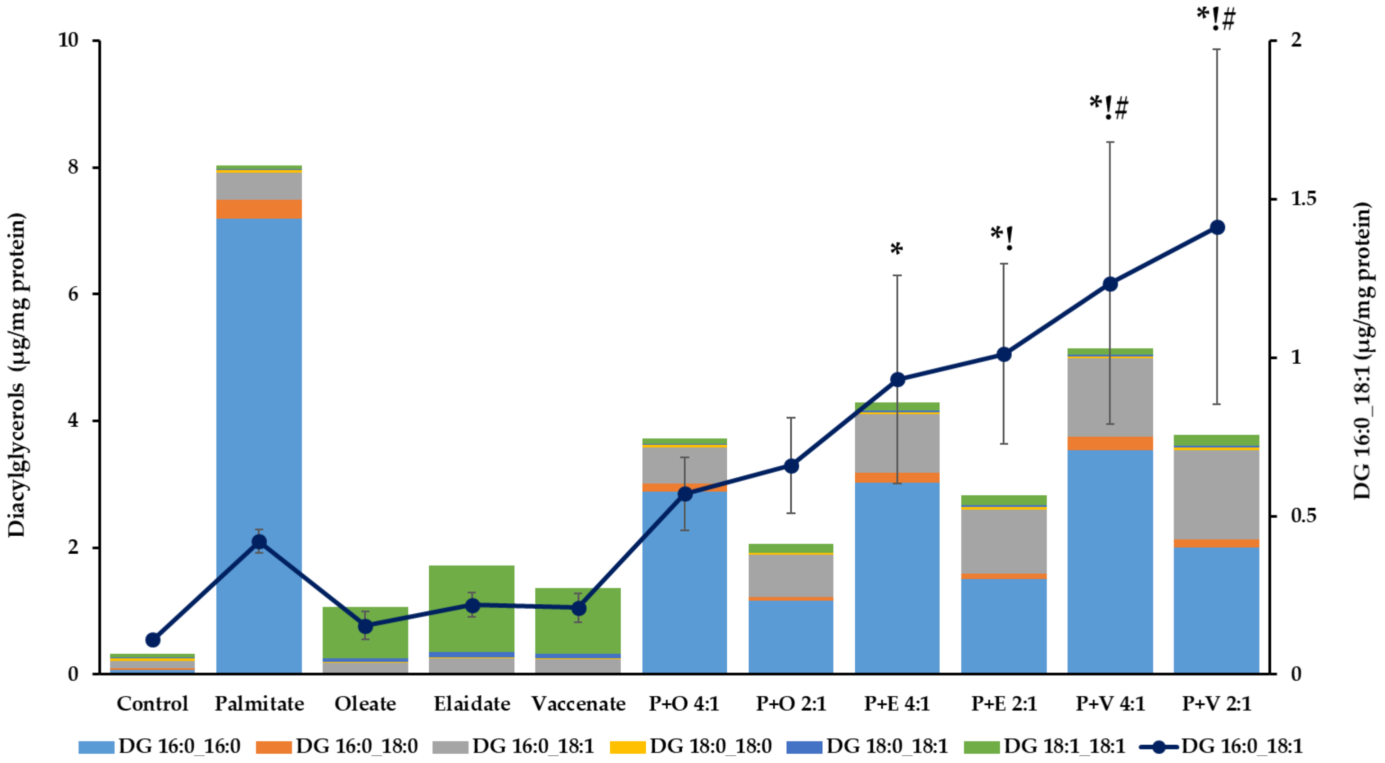

Figure S8. Diacylglycerol content of hepatoma cells in lipotoxicity after 4 h. Cells were treated with BSA-conjugated palmitate, oleate, elaidate or vaccenate (800 µM) or with palmitate (800 µM) and oleate, elaidate or vaccenate at 4:1 or 2:1 molar ratio (200 or 400 µM) at 70-80% confluence for 4 h. The amount of 6 relevant diacylglycerols (columns) was measured by LC-MS/MS. Data were normalized to the total protein content of the samples, and are shown as mean values of at least four independent experiments (see the mean and S.D. values in Table S3). DG 16:0_18:1 contents are also shown separately (line, right axis) as mean values ± S.D.; n≥4; statistically significant differences: *$P < 0.05$, v.s. BSA treated control; $^{!}P < 0.05$, v.s. palmitate treated samples; $^{\#}P < 0.05$, v.s. palmitate and oleate treated cells in case of combinational treatments at the corresponding ratio.

Table S7. Diacylglycerol content of hepatoma cells in lipotoxicity. Cells were treated with BSA-conjugated palmitate, oleate, elaidate or vaccenate (800 μM) or with palmitate (800 μM) and oleate, elaidate or vaccenate at 4:1 or 2:1 molar ratio (200 or 400 μM) at 70-80% confluence for 4 or 8 h. The amount of relevant diacylglycerols was measured by LC-MS/MS. Data were normalized to the total protein content of the samples, and are shown as mean values ± S.D.; n≥4.

Treatment (4 h)	Diacylglycerol content (ng/mg protein)					
	DG 16:0_16:0	DG 16:0_18:0	DG 16:0_18:1	DG 18:0_18:0	DG 18:0_18:1	DG 18:1_18:1
Control	67.0 ± 36.3	38.5 ± 34.1	110.5 ± 12.3	34.3 ± 27.8	15.3 ± 8.6	62.9 ± 16.2
Palmitate	7 193.3 ± 2 306.7	305.2 ± 81.7	420.5 ± 37.2	37.0 ± 20.7	12.8 ± 7.2	65.2 ± 11.1
cis-Oleate	24.0 ± 12.7	5.9 ± 4.8	153.9 ± 44.1	17.0 ± 9.0	52.1 ± 19.0	816.8 ± 176.4
trans-Elaidate	22.4 ± 7.2	7.9 ± 4.1	219.7 ± 37.9	18.9 ± 11.7	84.6 ± 36.6	1 369.3 ± 261.0
trans-Vaccenate	20.4 ± 5.1	8.1 ± 4.3	210.7 ± 45.3	20.8 ± 12.8	65.6 ± 29.7	1 037.9 ± 267.0
Palmitate + Oleate (4:1)	2 888.8 ± 973.4	126.9 ± 40.6	570.1 ± 116.1	35.1 ± 16.1	11.4 ± 3.1	94.8 ± 10.4
Palmitate + Oleate (2:1)	1 162.8 ± 414.0	65.7 ± 12.5	659.8 ± 151.4	28.2 ± 3.2	8.3 ± 0.2	135.5 ± 19.6
Palmitate + Elaidate (4:1)	3 032.2 ± 1 223.9	148.6 ± 43.6	931.4 ± 329.2	29.4 ± 12.9	22.8 ± 13.1	122.8 ± 25.3
Palmitate + Elaidate (2:1)	1 504.9 ± 550.8	90.5 ± 30.2	1 012.0 ± 283.4	33.2 ± 20.3	26.6 ± 13.7	163.5 ± 44.9
Palmitate + Vaccenate (4:1)	3 536.9 ± 1 296.1	211.2 ± 74.8	1 235.3 ± 443.9	33.3 ± 21.7	31.2 ± 24.3	99.9 ± 8.1
Palmitate + Vaccenate (2:1)	1 999.3 ± 817.4	133.3 ± 57.8	1 413.0 ± 560.4	30.2 ± 23.9	30.6 ± 16.0	174.5 ± 48.9

Treatment (8 h)	Diacylglycerol content (ng/mg protein)					
	DG 16:0_16:0	DG 16:0_18:0	DG 16:0_18:1	DG 18:0_18:0	DG 18:0_18:1	DG 18:1_18:1
Control	60.0 ± 23.4	23.4 ± 21.1	136.4 ± 46.5	33.7 ± 8.8	5.7 ± 1.5	58.2 ± 22.6
Palmitate	16 336.1 ± 2 395.0	740.4 ± 64.8	702.4 ± 131.1	81.3 ± 30.9	50.9 ± 26.9	63.8 ± 19.5
cis-Oleate	31.7 ± 19.7	20.5 ± 15.9	172.4 ± 63.9	39.6 ± 18.4	71.9 ± 32.5	1 178.4 ± 296.6
trans-Elaidate	34.4 ± 16.9	20.1 ± 11.8	249.7 ± 40.2	40.9 ± 15.1	142.3 ± 45.7	2 445.4 ± 293.7
trans-Vaccenate	33.3 ± 28.3	29.7 ± 20.2	288.2 ± 70.3	49.2 ± 9.7	134.0 ± 51.6	1 901.8 ± 287.4
Palmitate + Oleate (4:1)	7 174.8 ± 2 691.3	368.0 ± 123.2	1 089.4 ± 288.0	43.6 ± 20.4	41.1 ± 32.5	139.3 ± 42.5
Palmitate + Oleate (2:1)	1 946.5 ± 948.1	106.0 ± 50.8	983.5 ± 216.5	42.3 ± 13.9	17.7 ± 2.4	170.8 ± 39.1
Palmitate + Elaidate (4:1)	7 552.9 ± 2 447.8	436.6 ± 110.1	2 029.1 ± 550.5	40.7 ± 1.9	29.4 ± 3.5	167.7 ± 34.8
Palmitate + Elaidate (2:1)	3 201.3 ± 1 023.6	232.3 ± 76.3	1 921.1 ± 521.3	35.2 ± 14.1	43.3 ± 34.7	265.4 ± 72.3
Palmitate + Vaccenate (4:1)	7 882.0 ± 1 860.3	524.5 ± 133.1	2 539.3 ± 714.4	57.5 ± 18.7	53.4 ± 43.2	140.5 ± 33.9
Palmitate + Vaccenate (2:1)	3 941.7 ± 1 496.1	320.4 ± 137.3	2 726.5 ± 1 122.5	30.4 ± 5.2	60.2 ± 39.5	261.1 ± 111.6

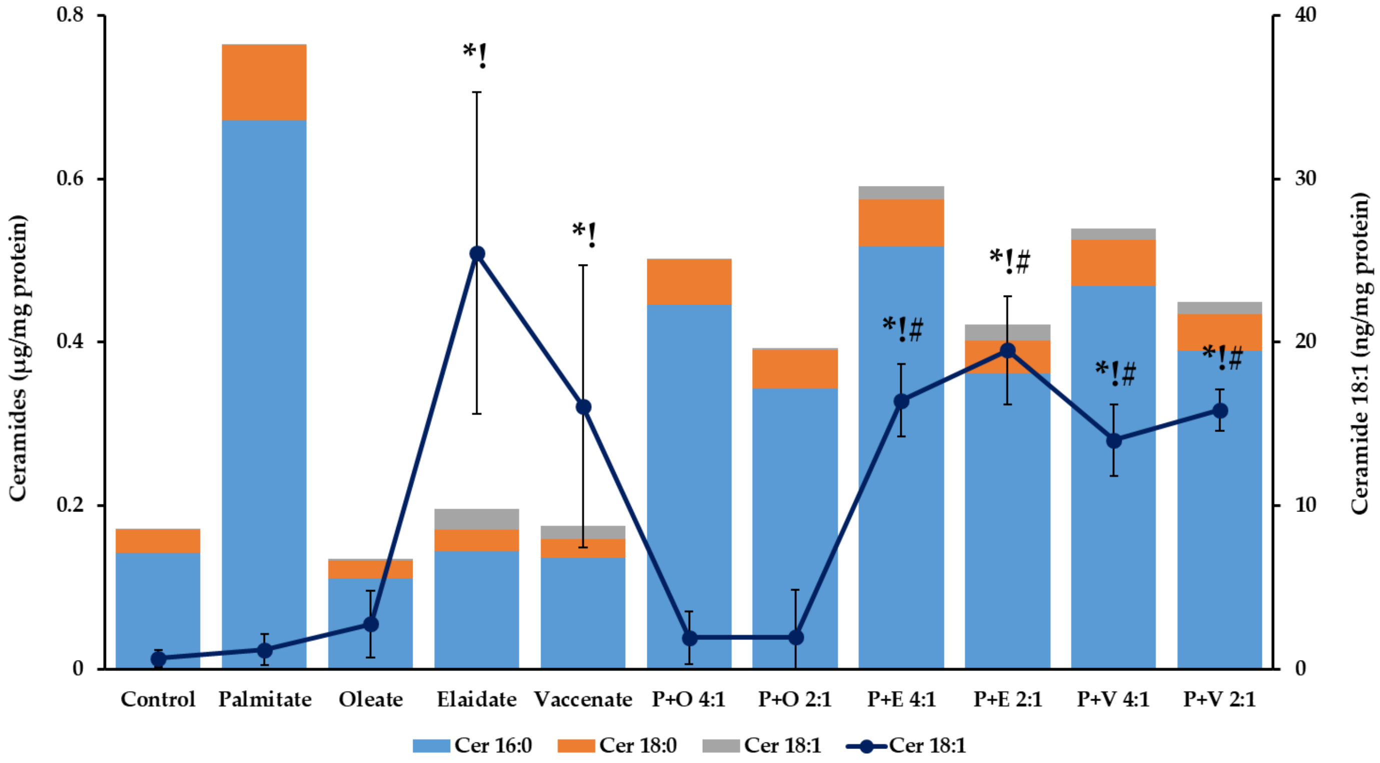

Figure S9. Ceramide content of hepatoma cells in lipotoxicity after 4 h. Cells were treated with BSA-conjugated palmitate, oleate, elaidate or vaccenate (800 µM) or with palmitate (800 µM) and oleate, elaidate or vaccenate at 4:1 or 2:1 molar ratio (200 or 400 µM) at 70-80% confluence, for 4 h. The amount of 3 relevant ceramides was measured by LC-MS/MS. Data were normalized to the total protein content of the samples, and are shown as mean values of at least four independent experiments (see the mean and S.D. values in Table S4). Ceramide 18:1 contents are also shown separately (line, right axis) as mean values ± S.D.; n≥3; statistically significant differences: *$P < 0.05$, v.s. BSA treated control; ![!]$P < 0.05$, v.s. palmitate treated samples; [#]$P < 0.05$, v.s. palmitate and oleate treated cells in case of combinational treatments at the corresponding ratio.

Table S8. Ceramide content of hepatoma cells in lipotoxicity. Cells were treated with BSA-conjugated palmitate, oleate, elaidate or vaccenate (800 μM) or with palmitate (800 μM) and oleate, elaidate or vaccenate at 4:1 or 2:1 molar ratio (200 or 400 μM) at 70-80% confluence for 4 or 8 h. The amount of 3 relevant ceramides was measured by LC-MS/MS. Data were normalized to the total protein content of the samples, and are shown as mean values ± S.D.; n≥3.

Treatment (4 h)	Ceramide content (ng/mg protein)		
	**Cer 16:0**	**Cer 18:0**	**Cer 18:1**
Control	142.3 ± 12.2	28.1 ± 0.8	0.6 ± 0.5
Palmitate	671.7 ± 182.9	92.4 ± 14.6	1.2 ± 0.9
cis-Oleate	110.5 ± 22.0	21.5 ± 2.6	2.8 ± 2.0
trans-Elaidate	143.6 ± 9.5	26.5 ± 3.3	25.4 ± 9.8
trans-Vaccenate	136.0 ± 19.7	23.6 ± 5.0	16.1 ± 8.6
Palmitate + Oleate (4:1)	445.8 ± 114.5	55.2 ± 11.6	1.9 ± 1.6
Palmitate + Oleate (2:1)	343.2 ± 80.2	47.9 ± 11.1	2.0 ± 2.9
Palmitate + Elaidate (4:1)	517.1 ± 117.8	57.8 ± 12.4	16.4 ± 2.2
Palmitate + Elaidate (2:1)	361.7 ± 81.7	40.4 ± 10.9	19.5 ± 3.3
Palmitate + Vaccenate (4:1)	468.8 ± 127.1	56.6 ± 10.0	14.0 ± 2.2
Palmitate + Vaccenate (2:1)	389.9 ± 107.1	44.2 ± 6.4	15.9 ± 1.2
**Treatment (8 h)**	**Ceramide content (ng/mg protein)**		
	**Cer 16:0**	**Cer 18:0**	**Cer 18:1**
Control	128.4 ± 43.1	19.9 ± 8.7	1.1 ± 1.3
Palmitate	1 769.5 ± 106.2	221.8 ± 18.1	2.5 ± 2.3
cis-Oleate	72.3 ± 19.8	13.1 ± 6.4	3.3 ± 2.5
trans-Elaidate	92.3 ± 30.3	14.6 ± 7.8	26.9 ± 8.8
trans-Vaccenate	107.9 ± 39.9	17.9 ± 7.5	18.8 ± 7.5
Palmitate + Oleate (4:1)	851.3 ± 534.3	93.1 ± 60.8	1.9 ± 1.7
Palmitate + Oleate (2:1)	391.2 ± 81.1	43.4 ± 7.1	0.9 ± 0.7
Palmitate + Elaidate (4:1)	1 023.8 ± 192.2	89.3 ± 6.4	33.3 ± 13.3
Palmitate + Elaidate (2:1)	582.7 ± 55.0	46.8 ± 6.1	30.2 ± 7.7
Palmitate + Vaccenate (4:1)	1 047.1 ± 420.2	98.3 ± 35.5	28.0 ± 13.7
Palmitate + Vaccenate (2:1)	678.4 ± 254.0	52.9 ± 16.5	28.6 ± 14.1

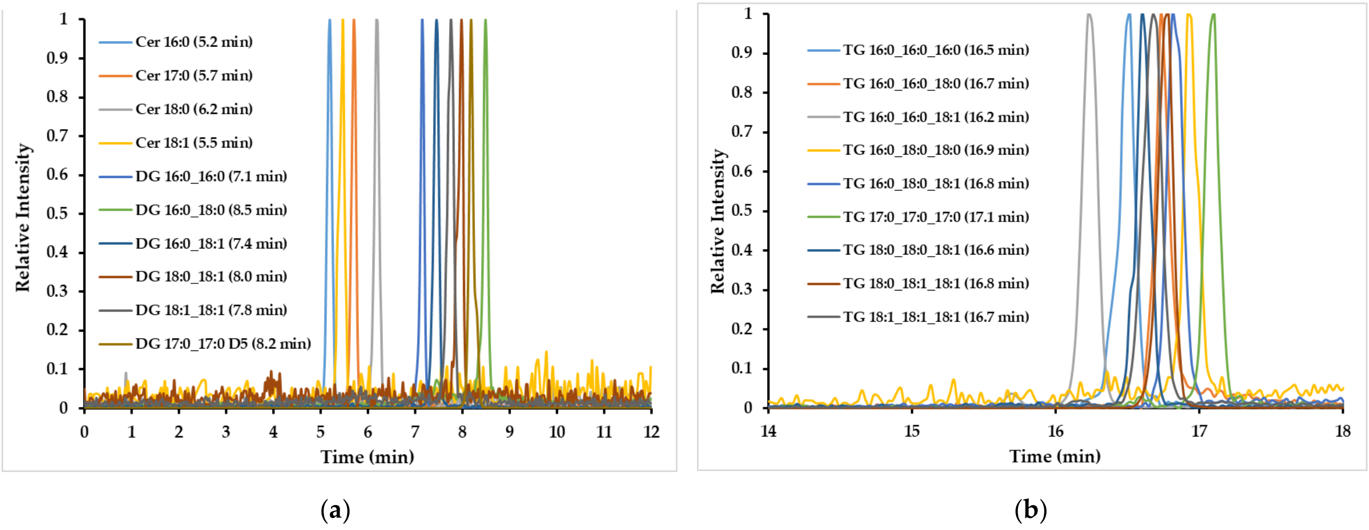

Figure S10. Representative chromatogram of the measured ceramides and diacylglycerols (**a**) and triacylglycerols (**b**) in a cell sample. The peaks are normalized to the corresponding peak maximums for each analyte due to the large difference between the analyte concentrations. Retention times are shown in parentheses.

Table S9. The m/z values of precursor and product ions for each analyte in mass spectrometry.

Analyte	Precursor ion	Product ion
Cer 17:0	552.0	264.2
Cer 17:0	552.0	534.0
Cer 16:0	538.6	264.2
Cer 16:0	538.6	520.6
Cer 18:0	566.5	264.2
Cer 18:0	566.5	548.6
Cer 18:1	564.6	264.2
Cer 18:1	564.6	546.5
DG 17:0_17:0 D5	619.4	332.2
DG 17:0_17:0 D5	619.4	584.4
DG 16:0_16:0	586.5	313.3
DG 16:0_18:0	614.6	341.3
DG 16:0_18:0	614.6	313.3
DG 16:0_18:1	612.5	339.3
DG 16:0_18:1	612.5	313.3
DG 18:0_18:0	642.6	341.3
DG 18:0_18:1	640.6	341.3
DG 18:0_18:1	640.6	339.3
DG 18:1_18:1	638.6	339.3
TG 17:0_17:0_17:0	866.4	579.4
TG 17:0_17:0_17:0	866.4	253.4
TG 16:0_16:0_18:1	850.8	577.5
TG 16:0_16:0_18:1	850.8	551.5
TG 16:0_16:0_16:0	824.5	551.5
TG 16:0_16:0_18:0	852.8	579.5
TG 16:0_16:0_18:0	852.8	551.5
TG 16:0_18:1_18:1	876.8	603.5
TG 16:0_18:1_18:1	876.8	577.5
TG 16:0_18:0_18:0	880.8	607.5
TG 16:0_18:0_18:0	880.8	579.5
TG 16:0_18:0_18:1	878.8	605.5
TG 16:0_18:0_18:1	878.8	579.5
TG 16:0_18:0_18:1	878.8	577.5
TG 18:1_18:1_18:1	902.8	603.5
TG 18:0_18:0_18:1	906.8	607.5
TG 18:0_18:0_18:1	906.8	605.5
TG 18:0_18:1_18:1	904.8	605.5
TG 18:0_18:1_18:1	904.8	603.5
TG 18:0_18:0_18:0	908.8	607.6
